# Correction: Pickering-emulsion-templated synthesis of 3D hollow graphene as an efficient oil absorbent

**DOI:** 10.1039/d1ra90143e

**Published:** 2021-09-17

**Authors:** Nurul Aqilah Pohan, Mohd Haniff Wahid, Zulkarnain Zainal, Nor Azowa Ibrahim

**Affiliations:** Department of Chemistry, Faculty of Science, Universiti Putra Malaysia 43400 Serdang Selangor Malaysia mw_haniff@upm.edu.my; Material Synthesis Laboratory, Institute of Advanced Technology, Universiti Putra Malaysia 43400 Serdang Selangor Malaysia

## Abstract

Correction for ‘Pickering-emulsion-templated synthesis of 3D hollow graphene as an efficient oil absorbent’ by Nurul Aqilah Pohan *et al.*, *RSC Adv.*, 2021, **11**, 3963–3971. DOI: 10.1039/D0RA09265G.

The author regrets that the funding information was incorrectly shown in the Acknowledgements section of the original manuscript. The corrected funding acknowledgements are as shown below.

The authors would like to thank the Ministry of Education of Malaysia for funding this study through grant no: FRGS/1/2018/STG01/UPM/02/11. Special thanks are extended to NanoMalaysia Berhad and Tex Cycle Technology (M) Berhad. Nurul Aqilah Pohan would like to thank the Ministry of Education of Malaysia for the Special Graduate Research Allowance Scheme.

The Royal Society of Chemistry apologises for these errors and any consequent inconvenience to authors and readers.

## Supplementary Material

